# A marker of biological age explains individual variation in the strength of the adult stress response

**DOI:** 10.1098/rsos.171208

**Published:** 2017-09-27

**Authors:** Clare Andrews, Daniel Nettle, Maria Larriva, Robert Gillespie, Sophie Reichert, Ben O. Brilot, Thomas Bedford, Pat Monaghan, Karen A. Spencer, Melissa Bateson

**Affiliations:** 1 Centre for Behaviour and Evolution, Institute of Neuroscience and Newcastle University Institute of Ageing, Newcastle University, Newcastle upon Tyne, UK; 2 School of Psychology and Neuroscience, University of St Andrews, St Andrews, UK; 3 School of Biological Sciences, Plymouth University, Plymouth, UK; 4 Institute of Biodiversity, Animal Health and Comparative Medicine, University of Glasgow, Glasgow, UK; 5 Department of Animal and Plant Sciences, University of Sheffield, Western Bank, Sheffield, UK

**Keywords:** stress response, corticosterone, biological age, telomere, *Sturnus vulgaris*, early-life adversity

## Abstract

The acute stress response functions to prioritize behavioural and physiological processes that maximize survival in the face of immediate threat. There is variation between individuals in the strength of the adult stress response that is of interest in both evolutionary biology and medicine. Age is an established source of this variation—stress responsiveness diminishes with increasing age in a range of species—but unexplained variation remains. Since individuals of the same chronological age may differ markedly in their pace of biological ageing, we asked whether biological age—measured here via erythrocyte telomere length—predicts variation in stress responsiveness in adult animals of the same chronological age. We studied two cohorts of European starlings in which we had previously manipulated the rate of biological ageing by experimentally altering the competition experienced by chicks in the fortnight following hatching. We predicted that individuals with greater developmental telomere attrition, and hence greater biological age, would show an attenuated corticosterone (CORT) response to an acute stressor when tested as adults. In both cohorts, we found that birds with greater developmental telomere attrition had lower peak CORT levels and a more negative change in CORT levels between 15 and 30 min following stress exposure. Our results, therefore, provide strong evidence that a measure of biological age explains individual variation in stress responsiveness: birds that were biologically older were less stress responsive. Our results provide a novel explanation for the phenomenon of developmental programming of the stress response: observed changes in stress physiology as a result of exposure to early-life adversity may reflect changes in ageing.

## 1. Introduction

The vertebrate stress response is highly conserved across taxa and functions to prioritize immediate survival over non-essential activities in the face of acute threats [1,2]. This change in priorities is mediated via the release of glucocorticoid hormones that temporarily suppress behaviour not critical to immediate survival, including foraging, self-maintenance, courtship, mating and parental care, while simultaneously promoting behaviour that aids avoiding, combating or escaping threats [3]. Given the importance of the stress response in orchestrating adaptive behaviour, it is interesting that there exists substantial between-individual variation in adult stress responsiveness [4]. Understanding the causes and consequences of this variation has been the goal of much recent research in both behavioural ecology and biomedicine [5–7]. One established source of variation in stress responsiveness is age: the acute stress response shows a decline with age in a range of species [4,8–13]. For example, a recent longitudinal study of hypothalamic–pituitary–adrenal (HPA) axis activity in house sparrows (*Passer domesticus*) showed that although baseline levels of the main avian glucocorticoid hormone, corticosterone (CORT), did not change with age, the level of CORT measured 30 min after exposure to a standardized acute stressor declined with age suggesting plasticity in the stress response [4]. However, despite the significant effect of age in this study, there remained substantial unexplained variation. One possible explanation for this variation is that it is future life expectancy rather than chronological age that determines behavioural priorities [14]. Although chronological age and future life expectancy are likely to be correlated, there can be considerable variation in the pace of ageing of individuals of the same chronological age resulting in different life expectancies [15–17]. The concept of biological age has been proposed to capture this variation in life expectancy [17], and we, therefore, propose that biological age could explain individual differences in the stress responsiveness of adult animals of the same chronological age.

The biological age of an individual can be objectively assessed by measuring biomarkers that typically decline with chronological age but that have been demonstrated to provide a better prediction of life expectancy than chronological age [17]. Telomere length (TL) has emerged as a candidate cellular biomarker of biological age in both humans and birds: as required, telomeres shorten with chronological age [16,18], and TL predicts longevity [16,19]. Exposure to adversity is believed to be central to biological ageing, with high levels of adversity in early life being particularly damaging [20]. In line with this expectation, telomeres are shortened by adversity in a range of species [21]. Specifically, there is mounting evidence from birds that developmental telomere attrition can be experimentally accelerated by early-life adversity [15,22,23], and that developmental telomere attrition is a stronger predictor of adult phenotypic outcomes, including survival, than a single cross-sectional measurement of adult TL [15,24,25].

In the current paper we study the relationships between developmental telomere attrition as a biomarker of biological age, and adult HPA axis function, in a relatively long-lived, iteroparous, passerine bird, the European starling (*Sturnus vulgaris*). In recent studies [22,23], we created two cohorts of starlings in which biological age was experimentally altered by manipulating early-life adversity. In the 2012 cohort of birds [22], quartets of siblings were cross-fostered either to broods of two chicks (low competition, LC), or broods of seven chicks (high competition, HC) between day 3 and day 15 post-hatching. In the 2013 cohort of birds [23], quartets of siblings were cross-fostered either to broods where they were approximately 5 g larger than their brood competitors (advantaged, ADV) or where they were approximately 5 g smaller (disadvantaged, DIS), between days 2 and 12. In both cases, the birds were brought into captivity at the end of the experimental manipulation and kept in uniform conditions until adulthood. In both cohorts, developmental telomere attrition measured in erythrocytes was accelerated in the higher competition group (HC and DIS [22,23]), although in the 2012 cohort this effect was restricted to the subset of HC birds that were smaller than their brood competitors. These cohorts provide a powerful method for examining developmental and familial origins of individual differences in adult stress responsiveness. First, the lives of the birds in the different experimental groups differed only during the brief, 10–12 day, period of the manipulation. Second, the use of cross-fostered siblings allows statistical estimation of the variance in adult stress responsiveness due to genetic and other parental effects, and that due to the subsequent manipulation of developmental experience and resulting biological ageing.

**Figure 1.**
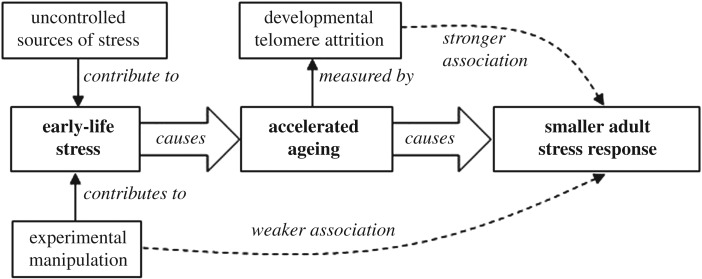
Hypothesized causal relationships and resulting predicted associations.

Here we report measurements of baseline plasma CORT, and the dynamics of the CORT response to an acute capture-handling-restraint stressor in the adult birds at approximately one year of age. We predicted that birds whose developmental telomere attrition, and hence biological ageing, had been accelerated by exposure to early-life adversity would respond to stress as adults as if they were older than their chronological age. Specifically, we predicted that birds with greater developmental telomere attrition would display an attenuated stress response, characterized by lower peak CORT levels and/or faster return to baseline CORT levels following exposure to a stressor. We did not predict effects of developmental telomere attrition on baseline CORT because previous studies of age and developmental adversity in birds do not typically report effects on baseline CORT [4,26,27]. We additionally predicted that if increased biological age causes a decline in adult stress responsiveness, then developmental telomere attrition, a biomarker of biological age, should be a stronger predictor of the size of the adult stress response than the developmental manipulation to which birds were exposed as chicks. This prediction emerges because developmental telomere attrition is hypothesized to be an integrative measure of the impact on the individual of all sources of developmental adversity. By contrast, the experimental treatment represents only one source of developmental adversity, and one by which different individuals might be affected to different extents depending on their stress resilience. Therefore, developmental telomere attrition should provide a superior proxy for early-life experience, and hence accelerated biological ageing, for use in explaining variation in adult stress responsiveness (figure 1). Finally, based on previous results from birds showing that developmental telomere attrition is a better predictor of life expectancy than telomere length [15], we predicted that developmental telomere attrition would also be a better predictor than adult telomere length of the size of the adult stress response.

## 2. Material and methods

### 2.1. Study animals and husbandry

Subjects were the two cohorts of European starlings whose history is described in detail in previous publications [22,23]. The 2012 cohort was aged 208–432 days at the time of stress response measurement, while the 2013 cohort was aged 428–456 days. In the period following fledging, birds lived in indoor aviaries with ad libitum food, except for periods in individual cages to complete behavioural experiments [24,25,28–31]. Time in cages was similar for all individuals. In both aviaries and cages 15 L : 9 D cycle was maintained from fledging onwards and resulted in the birds being photorefractory and in non-reproductive condition.

### 2.2. Telomere length and developmental telomere attrition

Two measures of TL were taken from each bird in order to derive a measure of developmental telomere attrition. The TLs used were from days 4 and 55 post-hatch for the 2012 cohort and days 3 and 12 for the 2013 cohort, for consistency with previous publications [24,25,31]. TL was measured by real-time quantitative PCR amplification of DNA extracted from erythrocytes [22,23]. The two TLs were used to obtain ΔTL, a standardized measure of developmental telomere attrition that corrects for the expected regression to the mean in repeated imperfectly correlated measurements [32]. A ΔTL value of zero represents the mean amount of developmental telomere attrition for that cohort, a negative number indicates greater than average attrition, while a positive number represents less than average attrition.

In addition to the developmental telomere attrition measurements, we also had measures of relative TL using the same methodology for the birds as adults of approximately one year old (TLy1; 2012 cohort, 427 days, as reported in Bateson *et al*. [31]; 2013 cohort, 389–406 days, previously unpublished data). We used the adult TLs to calculate telomere attrition between the earliest measurement and the one-year measurement, henceforth referred to as lifetime telomere attrition (lifeΔTL). Realized sample sizes for the telomere measurements were 23 birds and 33 birds for the 2012 and 2013 cohorts, respectively.

### 2.3. Blood sampling for corticosterone and assay

For CORT measurement, groups of up to eight birds (two natal families) were housed in individual cages with ad libitum food. For the 2012 birds, sampling was at the end of a behavioural experiment [24] meaning that birds had been in cages for a minimum of 28 days at the time of sampling. The 2013 birds were put into cages especially for this experiment and allowed to settle for a minimum of three nights prior to sampling. Body weight was measured around the time of blood sampling.

We used a standardized capture-handling-restraint protocol known to induce an acute stress response in starlings [33]. Between 14.00 and 15.30 on a given day we simultaneously sampled a randomly selected pair of siblings. The birds were not disturbed for two hours prior to sampling. The stressor began when the room lights were extinguished and two experimenters entered the room. Each experimenter caught one member of the pair, transferring the bird to an adjacent lit room. A baseline blood sample was taken within 3 min of the lights going off (mean time to baseline sample ± s.d., 86.1 ± 21.5 s (2012); 88.8 ± 20.4 s (2013)). Birds were then held in a cloth bag and further blood samples were taken at 15 and 30 min after the onset of the stressor. Blood sampling (approx. 120 µl per sample) was by puncture of the alar or metatarsal vein and collection using heparinized microcapillary tubes. Birds were returned to cages after the 30 min sample.

Blood samples were centrifuged to separate plasma, which was stored at −80°C until analysis. CORT was quantified in extracted plasma using a radioimmunoassay (RIA) that has been previously validated in starlings [34]. All blood samples were measured after extraction of 10–30 µl aliquots of plasma in 1 ml of ethyl diether by an RIA method [35] with [1,2,6,7-3H]-CORT. In 2012 the anti-CORT serum used was B3-163 (Esoterix, USA) and in 2013 we used anti-CORT serum ABIN880 (Antibodies Online). The cross reactivity data for B3-163 were: deoxycorticosterone 4%, cortisol less than 1%, aldosterone less than 1% and progesterone less than 1%; for ABIN880 the corresponding reactivities were: 1.5%, less than 0.1%, 0.2% and less than 0.1%. Comparison of these two anti-sera was undertaken, providing data that showed comparable CORT concentrations in a range of quality control samples (inter-antibody CVs at 25%, 50% and 75% binding were 3.4%, 5.2% and 3.6%, respectively). CORT levels were above the detection limit (2012: 0.24 ng ml^−1^; 2013: 0.04 ng ml^−1^) for all except seven samples at baseline; for these we inserted the detection limit of the assay as a conservative estimate of CORT levels. For all samples, extraction efficiency was individually estimated between 65% and 100% (mean 95.8%), with final CORT values being corrected accordingly for each sample. In 2012 samples were run in duplicate in a single assay (intra-assay coefficient of variation 19%), in 2013 we ran duplicates in three assays and intra-assay coefficients of variation were 7%, 5% and 7%. Inter-assay coefficient of variation across all four assays was 9% (calculated using standard curve data). CORT concentration at 50% binding averaged 1.15 ng ml^−1^. The data from two birds in the 2012 cohort were excluded due to concerns that the CORT assay was unreliable.

We derived three CORT variables previously used [26,27] to capture the dynamics of the birds' response to the stressor: (a) baseline CORT concentration (first sample); (b) peak CORT (higher of 15 and 30 min samples); (c) ΔCORT, the change in CORT between 15 and 30 min (calculated as CORT at 30 min − CORT at 15 min; ΔCORT is thus negative for an individual whose CORT levels reduce between 15 and 30 min and positive for an individual whose CORT continues to increase). Realized samples sizes for the CORT variables were: 2012, *n* = 29 for baseline CORT, *n* = 28 for peak CORT and ΔCORT; 2013, *n* = 36 for baseline CORT, *n* = 34 for peak CORT and ΔCORT.

### 2.4. Statistical analysis

Statistical analyses were conducted in R v. 3.3.2 [36]. We used general linear-mixed models implemented in ‘lme4’ [37]) including random intercepts for natal nest. Maximum-likelihood estimation was used unless otherwise stated. Significance testing for fixed effects was by likelihood-ratio test (LRT) using a criterion for significance of *p* < 0.05. The data and R script are publicly available via the Zenodo repository [38].

Where the outcome variable of the model was peak CORT, we included baseline CORT as a covariate, and where the outcome was ΔCORT, we included CORT at 15 min as a covariate. In the 2013 birds there was a significant relationship between ΔTL (our measure of developmental telomere attrition) and TL on day 3 (*B* = −0.18 ± 0.06; LRT 7.13, *p* = 0.008). For this cohort, we therefore included day 3 TL as an additional covariate in all models including ΔTL. In the 2012 cohort, the equivalent association (between ΔTL and day 4 TL) was absent (*B* = −0.12 ± 0.14, LRT = 0.64, *p* = 0.423), and we did not include starting TL in models involving ΔTL for this cohort.

In preliminary analysis, we tested whether any of the CORT variables was predicted by body condition (residual of body weight from tarsus length). Additionally, for the 2012 birds, where there was more variation in date of measurements, we tested whether age in days or number of days in cages predicted CORT variables. Finally, in both cohorts, we tested whether number of seconds between entering the room and obtaining the first sample predicted the baseline CORT value. Since none of these effects was significant (table 1), these variables are not included in the main analyses.

**Table 1.** Results of preliminary models predicting CORT variables. All models contain a random effect of natal family. For models predicting peak CORT, baseline CORT is entered as an additional predictor, and for those predicting ΔCORT, CORT at 15 min is included as an additional predictor.

cohort	CORT variable	fixed effects	LRT	*p*	*B* (s.e.)	*n*
2012	baseline CORT	body condition	1.03	0.310	−0.28 (0.27)	29
2012	peak CORT	body condition	3.08	0.079	−1.36 (0.71)	28
		baseline CORT	3.23	0.072	0.93 (0.50)	
2012	ΔCORT	body condition	1.50	0.220	−1.04 (0.84)	28
		CORT 15 min	2.57	0.109	−0.66 (0.38)	
2012	baseline CORT	age in days	0.24	0.622	0.01 (0.03)	29
2012	peak CORT	age in days	0.36	0.551	0.05 (0.08)	28
		baseline CORT	3.75	0.053	1.06 (0.52)	
2012	ΔCORT	age in days	0.02	0.894	0.01 (0.08)	28
		CORT 15 min	1.31	0.253	−0.47 (0.38)	
2012	baseline CORT	days in cages	0.06	0.806	0.07 (0.24)	29
2012	peak CORT	days in cages	0.07	0.797	0.22 (0.67)	28
		baseline CORT	3.90	0.048	1.08 (0.52)	
2012	ΔCORT	days in cages	0.01	0.924	−0.08 (0.74)	28
		CORT 15 min	1.21	0.272	−0.45 (0.38)	
2012	baseline CORT	seconds to obtain sample	0.23	0.631	−0.04 (0.08)	29
2013	baseline CORT	body condition	1.64	0.200	0.04 (0.03)	36
2013	peak CORT	body condition	0.07	0.797	−0.03 (0.11)	35
		baseline CORT	1.55	0.213	0.81 (0.64)	
2013	ΔCORT	body condition	1.69	0.194	−0.11 (0.08)	34
		CORT 15 min	3.75	0.053	−0.27 (0.13)	
2013	baseline CORT	seconds to obtain sample	0.65	0.422	0.01 (0.01)	36

We conducted a meta-analysis of the effects from the 2012 and 2013 cohorts using the R package ‘metafor’ [39]. For each cohort we calculated standardized effect sizes (standardized βs) and standard errors for the effect of ΔTL on baseline CORT, peak CORT and ΔCORT. We used fixed-effects models that allowed us to determine the sizes of the average true effects based on the data from the two cohorts that we had measured. In the analyses presented, the two cohorts are weighted equally, because the sample sizes were similar, but a weighted analysis produces the same qualitative outcomes.

## 3. Results

### 3.1. Descriptive statistics

Descriptive statistics for the CORT variables in the two cohorts are presented in table 2. In both cohorts a similar mean response to acute stress was observed: CORT levels were lowest at the baseline measurement, showed a large increase between baseline and 15 min following the start of the stressor and a smaller increase between 15 and 30 min. The majority of birds' CORT levels continued to rise between 15 and 30 min following the start of the stressor, but a substantial percentage fell in both cohorts (29% in 2012 and 47% in 2013; no significant difference between years; table 2). Absolute CORT levels at baseline, 15 and 30 min were all significantly higher in 2012 than 2013.

**Table 2.** Descriptive statistics for CORT variables in the two cohorts.

		CORT variable (mean ± s.d.) ng ml^−1^					
cohort	*n*^a^	baseline	15 min	30 min	peak CORT	ΔCORT	proportion of birds with ΔCORT < 0^b^
2012	28–29	17.32 ± 9.66	25.98 ± 15.22	37.16 ± 29.91	41.14 ± 29.35	11.19 ± 29.2	0.29
2013	34–36	2.2 ± 1.55	14.8 ± 5.18	15.06 ± 5.58	16.49 ± 5.10	0.37 ± 4.43	0.47
test statistic		8.34^c^	3.72^c^	3.86^c^	4.39^c^	1.94^c^	2.21^d^
*p*-value		<0.001	<0.001	<0.001	<0.001	0.062	0.137

^a^See text for exact *n* for each variable.

^b^ΔCORT < 0 signifies individuals whose CORT levels fell between 15 and 30 min.

^c^*t*-value from a Welch two-sample *t*-test.

^d^χ^2^ from 2 × 2 contingency table.

### 3.2. Familial components of corticosterone response

**Figure 2.**
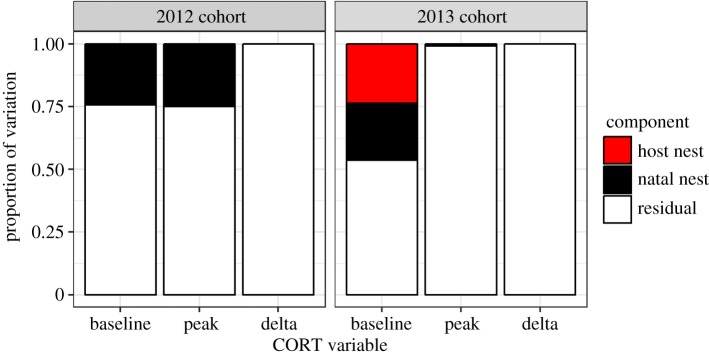
Components of variation (natal nest, host nest, residual) for each of the CORT measures in each of the two cohorts of cross-fostered birds.

To examine whether there were effects of natal nest (e.g. genetic, parental or very early environmental effects), or host nest, on each of our CORT variables, we conducted a variance components analysis. This entailed fitting a model with an intercept and nested random effects of natal nest and host nest for each CORT variable in turn using restricted maximum-likelihood estimation. In both cohorts, natal nest accounted for around 25% of the variation in baseline CORT (figure 2). Natal nest accounted for around 25% of the variation in peak CORT in the 2012 cohort, but only about 1% in the 2013 cohort. In 2013 only, host nest accounted for an additional 24% of the variation in baseline CORT. The natal and host nest component of ΔCORT was estimated at zero in both cohorts.

### 3.3. Experimental treatment and ΔTL as predictors of adult corticosterone

**Figure 3.**
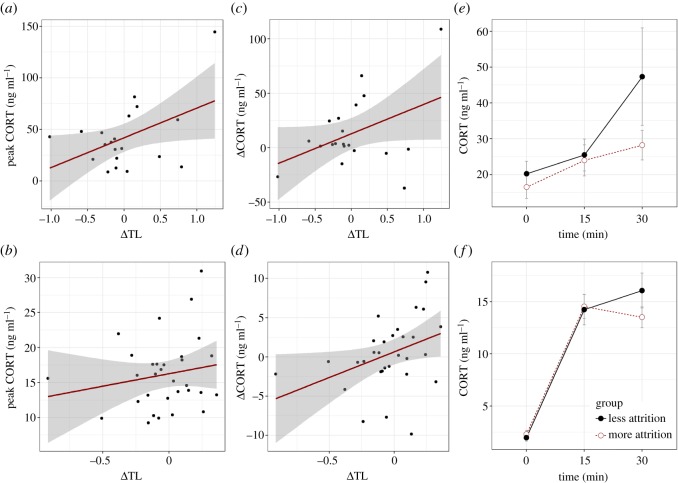
Summary of associations between developmental telomere attrition (ΔTL) and CORT variables. (*a*,*c*,*e*): 2012 cohort. (*b*,*d*,*f*): 2013 cohort. (*a*,*b*) Scatterplot of the association between ΔTL (more negative means greater attrition) and peak CORT. (*c*,*d*) Scatterplot of the association between ΔTL and ΔCORT. ΔCORT is the change in CORT between 15 and 30 min (where a negative value signifies a reduction in CORT). (*e*,*f*) Mean CORT at the three sample points for birds split at the median of ΔTL into those that experienced more (red open symbol, dotted line) and less developmental telomere attrition (black closed symbol, solid line). Error bars represent one standard error.

We ran a series of models predicting each adult CORT variable in each cohort from experimental treatment (HC versus LC for the 2012 cohort; DIS versus ADV for the 2013 cohort). In neither cohort were there significant relationships between experimental treatment and any of the CORT variables (table 3, models 1–6). We then replaced experimental treatment with ΔTL (i.e. developmental telomere attrition) as the main predictor variable (table 3, models 7–12). In neither cohort did ΔTL significantly predict baseline CORT. However, ΔTL significantly predicted peak CORT in the 2012 cohort; birds with greater telomere attrition had lower peak CORT (table 3 model 8; figure 3*a*). The effect of ΔTL on peak CORT was not significant in the 2013 birds, although the effect was in the same direction as that observed in 2012 (table 3 model 11; figure 3*b*). In both cohorts of birds, ΔTL significantly predicted ΔCORT (table 3 models 9 and 12; figure 3*c*,*d*). Although the pattern of CORT dynamics varied slightly between the two cohorts (figure 3*e*,*f*), in both cases, birds with more developmental telomere attrition showed a more negative change in CORT levels between 15 and 30 min (due either to a smaller increase or a greater decrease in CORT levels) compared with birds with less attrition.

**Table 3.** Output from linear-mixed models predicting CORT variables from experimental treatment and ΔTL. All models contain a random effect of natal family; for models predicting peak CORT, baseline CORT is entered as an additional predictor, and for those predicting ΔCORT, CORT at 15 min is included as an additional predictor; ΔTL is a standardized measure of developmental telomere attrition with a more negative value representing greater attrition; *p < 0.05.

model	cohort	CORT variable	fixed predictors	LRT	*p*	*B* (±s.e.)	*n*
1	2012	baseline	experimental treatment (HC versus LC)	0.34	0.559	−1.87 (3.18)	29
2	2012	peak	experimental treatment (HC versus LC)	0.01	0.920	−0.94 (9.34)	28
			baseline CORT	3.79	0.051	1.07 (0.53)	
3	2012	ΔCORT	experimental treatment (HC versus LC)	0.67	0.413	−8.48 (10.29)	28
			CORT 15 min	1.28	0.259	−0.44 (0.35)	
4	2013	baseline	experimental treatment (DIS versus ADV)	0.14	0.705	0.17 (0.44)	36
5	2013	peak	experimental treatment (DIS versus ADV)	0.05	0.818	0.39 (1.71)	34
			baseline CORT	0.84	0.360	0.51 (0.55)	
6	2013	ΔCORT	experimental treatment (DIS versus ADV)	1.89	0.169	−1.93 (1.39)	34
			CORT 15 min	3.47	0.063	−0.26 (0.14)	
7	2012	baseline	ΔTL	0.00	0.996	−0.02 (4.56)	21
8	2012	peak	ΔTL	5.95	0.015*	30.49 (11.05)	20
			baseline CORT	3.14	0.076	1.00 (0.53)	
9	2012	ΔCORT	ΔTL	4.31	0.038*	27.97 (12.74)	20
			CORT 15 min	1.64	0.200	−0.61 (0.46)	
10	2013	baseline	ΔTL	0.17	0.677	0.45 (1.05)	33
			TL day 3	0.13	0.713	0.14 (0.39)	
11	2013	peak	ΔTL	0.19	0.659	1.67 (3.77)	31
			TL day 3	1.36	0.243	−1.80 (1.47)	
			baseline CORT	0.52	0.472	0.45 (0.62)	
12	2013	ΔCORT	ΔTL	5.58	0.018*	7.73 (3.04)	31
			TL day 3	1.00	0.316	1.27 (1.22)	
			CORT 15 min	3.53	0.060	−0.28 (0.14)	

**Figure 4.**
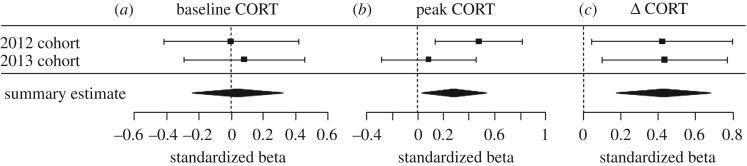
Forest plots showing estimated standardized β coefficients and their 95% confidence intervals for the effects of ΔTL on each of the CORT variables in both cohorts of birds. (*a*) Baseline CORT; (*b*) peak CORT; and (*c*) ΔCORT. For each CORT variable, the average summary estimate (±95% confidence intervals) for the two cohorts is shown as a black diamond. Zero represents no association. Note that for peak CORT and ΔCORT the diamond does not cross zero, indicating significant average effects.

In order to aggregate the results from the two cohorts of birds and estimate the sizes of the average true effects of ΔTL on the three CORT variables, we conducted a formal meta-analysis of the two experiments. Figure 4 shows forest plots with summary estimates of the effects for baseline CORT, peak CORT and ΔCORT. For baseline CORT the summary estimate effect size does not differ significantly from zero, whereas for both peak CORT and ΔCORT the summary estimates differ from zero: birds with greater telomere attrition had lower peak CORT and a more negative ΔCORT, indicating an attenuated stress response.

### 3.4. ΔTL versus other telomere-related biomarkers as predictors of adult corticosterone

As well as ΔTL, we had two other telomere-related variables for each bird, namely adult TL at one year (TLy1), and telomere attrition over the bird's lifetime to that point (lifeΔTL). These alternative measures were all positively correlated (2012 birds: ΔTL and TLy1, *r*_19_ = 0.44; ΔTL and lifeΔTL: *r*_19_ = 0.53; TLy1 and lifeΔTL, *r*_19_ = 0.92; 2013 birds: ΔTL and TLy1, *r*_31_ = 0.06; ΔTL and lifeΔTL: *r*_31_ = 0.61; TLy1 and lifeΔTL, *r*_31_ = 0.38) meaning that they should not be entered into the same model as predictors. To establish whether ΔTL was the best telomere-related predictor of adult CORT variables, for the cases where ΔTL significantly predicted an adult CORT variable (i.e. ΔCORT in both cohorts and peak CORT in the 2012 cohort), we compared the fit of models in which ΔTL was replaced by TLy1 or lifeΔTL. Model comparison was done using the R package ‘AICcmodavg’ [40] and a modified version of Akaike's information criterion (AICc) recommended for small sample sizes [41]. In each case, the best-fitting model (lowest AICc) was that using ΔTL as the predictor (table 4). Evidence ratios [41] showed that the best-fitting model was between 2.64 and 19.54 times more likely to be the best-approximating model than the alternatives with either TLy1 or lifeΔTL as predictors.

**Table 4.** Comparison of model fits (AICc) and evidence ratios for CORT variables showing a significant association with developmental telomere attrition, using alternative telomere-related predictors. All models contain a random effect of natal family; for models predicting peak CORT, baseline CORT is entered as an additional predictor, and for those predicting ΔCORT, CORT at 15 min is included as an additional predictor.

cohort	CORT variable	fixed predictors^a^	AICc	ΔAICc	evidence ratio
2012	peak	ΔTL + baseline CORT	199.64	0	
		TLy1 + baseline CORT	205.44	5.81	18.27
		lifeΔTL + baseline CORT	205.58	5.94	19.54
	ΔCORT	ΔTL + CORT 15 min	204.40	0	
		TLy1 + CORT 15 min	208.33	3.93	7.13
		lifeΔTL + CORT 15 min	208.71	4.31	8.63
2013	ΔCORT	ΔTL + TL day 3 + CORT 15 min	186.72	0	
		TLy1 + CORT 15 min	188.66	1.94	2.64
		lifeΔTL + TL day 3 + CORT 15 min	190.98	4.26	8.42

^a^ΔTL, standardized measure of developmental telomere attrition with a more negative value representing greater developmental telomere attrition; TLy1, TL at 1 year; lifeΔTL, standardized measure of change in TL over the lifetime thus far with a more negative value representing greater telomere attrition.

## 4. Discussion

Using two cohorts of adult European starlings that had previously been subjected to an experimental manipulation of early-life adversity, we tested the hypothesis that the rate of biological ageing, measured via the extent to which erythrocyte telomeres had shortened during development (developmental telomere attrition, ΔTL), explained individual variation in adult stress responsiveness. We predicted that birds whose developmental telomere attrition, and hence biological ageing, had been accelerated by exposure to early-life adversity would respond to stress as adults as if they were older than their chronological age, and hence show an attenuated response to an acute stressor. Our results supported this prediction: aggregating the results from both cohorts, we found that birds who had undergone greater developmental telomere attrition had lower peak CORT levels and more negative ΔCORT (i.e. either a smaller increase in CORT or a greater decrease in CORT between 15 and 30 min following the start of the stressor), resulting in an attenuated acute stress response. We found no relationship between developmental telomere attrition and baseline CORT in either cohort, and 25% of the variation in baseline CORT was familial. We also predicted that if increased biological age is responsible for variation in the adult stress response, then, developmental telomere attrition should be a stronger predictor of the size of the stress response than the manipulation of adversity to which the birds were exposed as chicks. In support of this prediction, in neither cohort were there significant effects of the experimental manipulation itself on any of the adult birds' CORT variables. Finally, we predicted that developmental telomere attrition would be a better predictor than adult telomere length of the size of the adult stress response. In both cohorts, we found that developmental telomere attrition was a better predictor of adult HPA axis function (peak and/or ΔCORT) than either of two alternative biomarkers of biological age, namely lifetime telomere attrition (lifeΔTL) and adult TL.

Before discussing the consistent results emerging from our two cohorts outlined above, we first address the differences. There were substantial differences in the absolute values of the CORT levels between our two cohorts: all of the CORT levels (baseline, 15 and 30 min) were higher in the 2012 cohort (table 2). We are hesitant to over-interpret these differences, because we imposed different developmental manipulations and used slightly different procedures immediately prior to CORT sampling and for the CORT assays in the two cohorts, meaning that the differences could be due to several factors. Although the differences in CORT at 15 and 30 min were in the direction that would be expected given that the 2013 birds were on average 122 days (38%) older than the 2012 birds at the time of the CORT measurements, the lack of overlap between the CORT levels in the two years suggests that the age difference between the cohorts is unlikely to be the only explanation for the CORT difference. Furthermore, we found no effect of biological age on baseline CORT, and baseline CORT was higher in 2012 than 2013. An alternative (or additional) explanation for the lower CORT levels in 2013 is that the birds had only been in cages for a minimum of three days prior to sampling (compared with 28 days in 2012) and thus may not have fully acclimated to the cages at the time of sampling. Lack of acclimation could potentially have resulted in a temporary decrease in adrenal capacity to produce CORT (adrenal insufficiency), explaining the lower CORT levels in all samples in 2013.

The lack of a significant effect of the experimental manipulations on adult CORT variables may appear perplexing given that our previous data showed that the manipulations caused changes in developmental telomere attrition in both cohorts [22,23], and that we have shown here that developmental telomere attrition predicts adult CORT variables. However, although developmental telomere attrition was related to experimental treatment group membership in both cohorts, there was some additional variation in developmental telomere attrition not attributable to the experimental manipulations. Given our model (figure 1), it is, therefore, to be expected that associations between the experimental manipulations and adult CORT variables should be weaker than those between developmental telomere attrition and adult CORT variables. Although none of the CORT variables were significantly predicted by the experimental manipulation, in both cohorts the birds in the higher stress group (HC in 2012 and DIS in 2013) had lower peak CORT and more negative ΔCORT, consistent with weaker associations in the same direction as those found in the statistical models with developmental telomere attrition as predictor (see parameter estimates in table 3). Thus, there is no conflict within our results, and our findings are compatible with the model depicted in figure 1.

Our main finding, that a marker of biological age (developmental telomere attrition) explains adult stress responsiveness, is correlational. This raises questions about whether the association could be the product of a third, uncontrolled, variable rather than being causal. However, the fact that we experimentally generated at least some of the variation in biological age, by manipulating the birds’ early-life experience [22,23], reduces the likely involvement of a third variable. Furthermore, we were able to replicate the same basic pattern in two different cohorts that differed in a number of respects. We, therefore, believe that our results offer strong support for a direct role of biological age in determining the size of adult stress response in starlings.

We analysed two different metrics of the acute stress response: peak CORT, which was the highest level of CORT recorded following the stressor, and ΔCORT, which was the change in CORT between 15 and 30 min following the onset of the stressor. ΔCORT has previously been interpreted as a measure of negative feedback within the HPA axis, with a more negative or less positive value being indicative of stronger negative feedback [26,27,42]. However, a more negative value of ΔCORT could additionally be caused by reductions in: corticotropin releasing hormone (CRH) and/or arginine vasotocin (AVT) from the hypothalamus, pituitary sensitivity to CRH and/or AVT, the ability of the pituitary to produce adrenocorticotropin (ACTH), adrenal sensitivity to ACTH and/or ability of the adrenal gland to produce CORT [43]. It is also possible that more negative ΔCORT could result from faster acclimation to the ongoing stress of bag restraint. Thus while it is safe to conclude that the biologically older birds demonstrated an attenuated stress response, characterized by lower CORT production overall, further work is needed to identify the mechanism or mechanisms underlying this.

Our results are compatible with results on changes in CORT variables with increasing chronological age from a range of bird species. These typically show no effects of age on baseline CORT, but less elevated CORT levels some minutes after a standardized stressor in chronologically older birds [4,8,9,11]. Although some studies of birds report an inverted U-shaped relationship between the magnitude of the stress response and chronological age, the secondary increase in the size of the stress response in these studies occurs only in the very oldest individuals [8,10,12]. Since starlings have a maximum longevity of 22.9 years [44], and the birds in the current study were under two years old at the time of the measurements, we would not have expected to see an increase in stress responsiveness associated with old (chronological or biological) age.

Two explanations exist for the observed relationship between increased chronological or biological age and reduced stress responsiveness. A senescence hypothesis suggests that reduced stress responsiveness is simply a pathological consequence of the accumulated somatic damage that constitutes biological ageing. By contrast, an adaptive hypothesis suggests that reduced stress responsiveness is a strategic response to increased biological age and consequent reduced life expectancy. Evolutionary logic suggests that iteroparous animals should behave differently as they age, shifting priorities towards activities likely to promote immediate reproductive success and away from future survival as remaining life expectancy declines [45] (with a possible reversal in very old age when ‘terminal restraint’ could be adaptive [10]). Previous authors have suggested that age-related changes in CORT responsiveness are likely to be adaptive rather than simply resulting from a reduced capacity to produce CORT resulting from senescence in the HPA axis [4,9]. However, the evidence supporting this claim is weak, being predominantly based on the lack of changes in baseline CORT with increasing age and evidence for increased reproductive performance with age in common terns (*Sterna hirundo*) [9]. The house sparrow data described above [4] do not eliminate a pathological decline in capacity to mount a stress response due to senescence within the HPA axis. Only two blood samples were taken following the onset of the stressor (baseline and 30 min) providing no information on whether the lower levels of CORT at 30 min in the older birds were due to reduced release of CORT resulting in a lower peak response, or to faster recovery of CORT baseline levels, or to both effects. Evidence for faster negative feedback with age would constitute stronger support for the adaptive account of the age-related changes, because senescence is widely held to involve progressive damage to cellular mechanisms, thus *enhanced* functioning of feedback mechanisms would be unlikely to result from senescence [46]. Our finding that ΔCORT was more negative in birds with greater biological age strengthens the adaptive account somewhat, because this result is compatible with stronger negative feedback within the HPA axis. However, our results do not rule out a senescence hypothesis due to the multiple possible interpretations of ΔCORT discussed above. Further studies investigating how HPA responses to ACTH stimulation and dexamethasone suppression change with chronological and biological age would be informative.

Our discussion suggests a new perspective on the phenomenon of ‘developmental programming’ of the stress response, whereby early-life events lead to changes in the adult phenotype [47]. It is well established in both mammals and birds that exposure to early-life adversity of various kinds has developmental programming effects on adult HPA axis function [47]. The nature and direction of these developmental programming effects varies depending on the species, the type and timing of the stressor, and the age at manipulation and measurement [27,48–51]. The hypothesis that some of this variation might be adaptive has been extensively discussed, but there is currently no consensus over whether, and if so, how these effects are adaptive [2].

Developmental programming of adult HPA axis function has often been explained with reference to the predictive adaptive response (PAR) hypothesis, which suggests that adversity experienced in early life provides a ‘weather forecast’ to the developing animal regarding the frequency and magnitude of stressors likely in its future environment [52]. The animal responds to this information by developing a stress response that will be adaptive in the predicted adult environment. Whether developmental programming effects represent adaptive matching of the individual to its likely adult environment is debatable [53,54]. From a theoretical perspective, the PAR idea may be more applicable to short-lived animals than longer-lived animals, such as primates and some birds, for which the correlation between the developmental and adult environments is likely to be weak or non-existent [53,55]. Moreover, our current data do not fit well with the PAR hypothesis. In both cohorts, developmental telomere attrition (a measure of state) was a better predictor of the adult stress response than the experimental treatment to which the chicks were exposed during early life (a potential source of information about the future environment). Therefore, our results are more compatible with an account of developmental plasticity whereby the birds are responding to a change in their state (in this case biological age) as opposed to using information that they have received as nestlings to predict their adult environment. We are currently agnostic over whether the response to a change in state is simply a symptom of senescence, or whether it could be an adaptive response to a shorter predicted lifespan [53].

If, as we argue, developmental adversity hastens biological ageing and biological age influences adult stress responsiveness, then we predict that developmental adversity should be associated with an attenuated adult stress response more generally. There have been many studies of the effects of various forms of pre- and post-hatching developmental stress on subsequent stress responsiveness in birds. In accord with our predictions, Zimmer *et al*. [26] found an attenuated CORT response to an acute stressor in sexually mature Japanese quail (*Coturnix japonica*) that had been exposed to CORT injections prior to hatching. However, apparently against our predictions, previous studies in starlings [34,51], zebra finches (*Taeniopygia guttata*) [27,48] and European shags (*Phalacrocorax aristotelis*) [56] all show lower ΔCORT and/or higher peak CORT in developmentally stressed birds. A possible explanation for these different results lies in the age at which the acute stress response was measured. In the current study and that of Zimmer *et al*. [26] CORT responses were measured in sexually mature adult birds, whereas in the other studies (which find the reverse association) CORT responses were measured in nestlings or juveniles. In line with this hypothesis, Crino *et al*. [48] found that the effect of developmental stress on ΔCORT in zebra finches had disappeared, though in their case had not reversed, once their birds were adults. They concluded that effects of developmental stress on CORT responsiveness in young animals could be reinterpreted as a reaction to current or recent stress as opposed to developmental programming of the adult stress response (see also [51]).

As a final point, our study is novel in showing that baseline and peak CORT have a moderate natal familial component (23–24% of the variance in baseline CORT and 1–25% of the variance in peak CORT was explained by natal nest), while ΔCORT, the variable that most often demonstrates developmental programming effects in birds, does not. Although findings from other species of mammals and birds show that individual differences in CORT responsiveness to stress have a genetic component [57,58], these studies typically do not distinguish between peak CORT and ΔCORT, both of which contribute to CORT responsiveness. Our findings are, therefore, compatible with these previous results, but extend them by providing evidence that ΔCORT is not strongly familial.

## Data accessibility

The raw data and R script from this experiment are publicly available at: https://doi.org/10.5281/zenodo.846830 [38].

## References

[1] Wingfield JC, Sapolsky RM 2003 Reproduction and resistance to stress: when and how. J. Neuroendocrinol. 15, 711–724. (doi:10.1046/j.1365-2826.2003.01033.x) 12834431 10.1046/j.1365-2826.2003.01033.x

[2] Monaghan P, Spencer KA 2014 Stress and life history. Curr. Biol. 24, R408–R412. (doi:10.1016/j.cub.2014.04.017) 24845673 10.1016/j.cub.2014.04.017

[3] Sapolsky RM, Romero LM, Munck AU 2000 How do glucocorticoids influence stress responses? Integrating permissive, suppressive, stimulatory and preparative actions. Endocr. Rev. 21, 55–89. (doi:10.1210/er.21.1.55) 10696570 10.1210/edrv.21.1.0389

[4] Lendvai Z, Giraudeau M, Bókony V, Angelier F, Chastel O 2015 Within-individual plasticity explains age-related decrease in stress response in a short-lived bird. Biol. Lett. 11, 20150272 (doi:10.1098/rsbl.2015.0272) 26179799 10.1098/rsbl.2015.0272 PMC4528442

[5] Monaghan P 2014 Organismal stress, telomeres and life histories. J. Exp. Biol. 217, 57–66. (doi:10.1242/jeb.090043) 24353204 10.1242/jeb.090043

[6] Romero LM 2004 Physiological stress in ecology: lessons from biomedical research. Trends Ecol. Evol. 19, 249–255. (doi:10.1016/j.tree.2004.03.008) 16701264 10.1016/j.tree.2004.03.008

[7] Seckl JR 2004 Prenatal glucocorticoids and long-term programming. Eur. J. Endocrinol. 151(Suppl), U49–U62. (doi:10.1530/eje.0.151U049) 15554887 10.1530/eje.0.151u049

[8] Wilcoxen TE, Boughton RK, Bridge ES, Rensel MA, Schoech SJ 2011 Age-related differences in baseline and stress-induced corticosterone in Florida scrub-jays. Gen. Comp. Endocrinol. 173, 461–466. (doi:10.1016/j.ygcen.2011.07.007) 21827761 10.1016/j.ygcen.2011.07.007

[9] Heidinger BJ, Nisbet ICT, Ketterson ED 2006 Older parents are less responsive to a stressor in a long-lived seabird: a mechanism for increased reproductive performance with age? Proc. R. Soc. B 273, 2227–2231. (doi:10.1098/rspb.2006.3557) 10.1098/rspb.2006.3557 PMC1635515 16901843

[10] Elliott KH, O'Reilly KM, Hatch SA, Gaston AJ, Hare JF, Anderson WG 2014 The prudent parent meets old age: a high stress response in very old seabirds supports the terminal restraint hypothesis. Horm. Behav. 66, 828–837. (doi:10.1016/j.yhbeh.2014.11.001) 25448533 10.1016/j.yhbeh.2014.11.001

[11] Heidinger BJ, Chastel O, Nisbet ICT, Ketterson ED 2010 Mellowing with age: older parents are less responsive to a stressor in a long-lived seabird. Funct. Ecol. 24, 1037–1044. (doi:10.1111/j.1365-2435.2010.01733.x)

[12] López-Jiménez L, Blas J, Tanferna A, Cabezas S, Marchant T, Hiraldo F, Sergio F 2017 Lifetime variation in feather corticosterone levels in a long-lived raptor. Oecologia 183, 315–326. (doi:10.1007/s00442-016-3708-0) 27568027 10.1007/s00442-016-3708-0

[13] Jessop TS, Hamann M 2005 Interplay between age class, sex and stress response in green turtles (*Chelonia mydas*). Aust. J. Zool. 53, 131–136. (doi:10.1071/ZO04061)

[14] McNamara JM, Houston AI, Barta Z, Scheuerlein A, Fromhage L 2009 Deterioration, death and the evolution of reproductive restraint in late life. Proc. R. Soc. B 276, 4061–4066. (doi:10.1098/rspb.2009.0959) 10.1098/rspb.2009.0959 PMC2825774 19726476

[15] Boonekamp J, Mulder G, Salomons H, Dijkstra C, Verhulst S 2014 Nestling telomere shortening, but not telomere length, reflects developmental stress and predicts survival in wild birds. Proc. R. Soc. B 281, 20133287 (doi:10.1098/rspb.2013.3287) 10.1098/rspb.2013.3287 PMC4024283 24789893

[16] Heidinger BJ, Blount JD, Boner W, Griffiths K, Metcalfe NB, Monaghan P 2012 Telomere length in early life predicts lifespan. Proc. Natl Acad. Sci. USA 109, 1743–1748. (doi:10.1073/pnas.1113306109) 22232671 10.1073/pnas.1113306109 PMC3277142

[17] Levine ME 2013 Modeling the rate of senescence: can estimated biological age predict mortality more accurately than chronological age? J. Gerontol. A Biol. Sci. Med. Sci. 68, 667–674. (doi:10.1093/gerona/gls233) 23213031 10.1093/gerona/gls233 PMC3660119

[18] Hastie ND, Dempster M, Dunlop MG, Thompson AM, Green DK, Allshire RC 1990 Telomere reduction in human colorectal carcinoma and with ageing. Nature 346, 866–868. (doi:10.1038/346866a0) 2392154 10.1038/346866a0

[19] Kimura M, et al. 2008 Telomere length and mortality: a study of leukocytes in elderly Danish twins. Am. J. Epidemiol. 167, 799–806. (doi:10.1093/aje/kwm380.Telomere) 18270372 10.1093/aje/kwm380 PMC3631778

[20] Danese A, McEwen BS 2012 Adverse childhood experiences, allostasis, allostatic load, and age-related disease. Physiol. Behav. 106, 29–39. (doi:10.1016/j.physbeh.2011.08.019) 21888923 10.1016/j.physbeh.2011.08.019

[21] Bateson M 2016 Cumulative stress in research animals: telomere attrition as a biomarker in a welfare context? Bioessays 38, 201–212. (doi:10.1002/bies.201500127) 26645576 10.1002/bies.201500127 PMC4737400

[22] Nettle D, Monaghan P, Boner W, Gillespie R, Bateson M 2013 Bottom of the heap: having heavier competitors accelerates early-life telomere loss in the European starling, *Sturnus vulgaris*. PLoS ONE 8, e83617 (doi:10.1371/journal.pone.0083617) 24386235 10.1371/journal.pone.0083617 PMC3873947

[23] Nettle D, Monaghan P, Gillespie R, Brilot B, Bedford T, Bateson M 2015 An experimental demonstration that early-life competitive disadvantage accelerates telomere loss. Proc. R. Soc. B 282, 20141610 (doi:10.1098/rspb.2014.1610) 10.1098/rspb.2014.1610 PMC4262165 25411450

[24] Bateson M, Brilot BO, Gillespie R, Monaghan P, Nettle D 2015 Developmental telomere attrition predicts impulsive decision-making in adult starlings. Proc. R. Soc. B. 282, 20142140 (doi:10.1098/rspb.2014.2140) 10.1098/rspb.2014.2140 PMC4286045 25473012

[25] Nettle D, Andrews CP, Monaghan P, Brilot BO, Bedford T, Gillespie R, Bateson M 2015 Developmental and familial predictors of adult cognitive traits in the European starling. Anim. Behav. 107, 239–248. (doi:10.1016/j.anbehav.2015.07.002) 26405302 10.1016/j.anbehav.2015.07.002 PMC4550429

[26] Zimmer C, Boogert NJ, Spencer KA 2013 Developmental programming: cumulative effects of increased pre-hatching corticosterone levels and post-hatching unpredictable food availability on physiology and behaviour in adulthood. Horm. Behav. 64, 494–500. (doi:10.1016/j.yhbeh.2013.07.002) 23891687 10.1016/j.yhbeh.2013.07.002 PMC3791420

[27] Spencer KA, Evans NP, Monaghan P 2009 Postnatal stress in birds: a novel model of glucocorticoid programming of the hypothalamic-pituitary-adrenal axis. Endocrinology 150, 1931–1934. (doi:10.1210/en.2008-1471) 19095740 10.1210/en.2008-1471

[28] O'Hagan D, Andrews CP, Bedford T, Bateson M, Nettle D 2015 Early life disadvantage strengthens flight performance trade-offs in European starlings, *Sturnus vulgaris*. Anim. Behav. 102, 141–148. (doi:10.1016/j.anbehav.2015.01.016) 25843958 10.1016/j.anbehav.2015.01.016 PMC4370370

[29] Andrews C, Viviani J, Egan E, Bedford T, Brilot B, Nettle D, Bateson M 2015 Early life adversity increases foraging and information gathering in European starlings. Anim. Behav. 109, 123–132. (doi:10.1016/j.anbehav.2015.08.009) 26566292 10.1016/j.anbehav.2015.08.009 PMC4615135

[30] Bloxham L, Bateson M, Bedford T, Brilot B, Nettle D 2014 The memory of hunger: developmental plasticity of dietary selectivity in the European starling, *Sturnus vulgaris*. Anim. Behav. 91, 33–40. (doi:10.1016/j.anbehav.2014.02.025) 24910465 10.1016/j.anbehav.2014.02.025 PMC4045381

[31] Bateson M, Emmerson M, Ergün G, Monaghan P, Nettle D 2015 Opposite effects of early-life competition and developmental telomere attrition on cognitive biases in juvenile European starlings. PLoS ONE 10, e0132602 (doi:10.1371/journal.pone.0132602) 26222390 10.1371/journal.pone.0132602 PMC4519284

[32] Verhulst S, Aviv A, Benetos A, Berenson GS, Kark JD 2013 Do leukocyte telomere length dynamics depend on baseline telomere length? An analysis that corrects for ‘regression to the mean’. Eur. J. Epidemiol. 28, 859–866. (doi:10.1007/s10654-013-9845-4) 23990212 10.1007/s10654-013-9845-4

[33] Rich EL, Romero LM 2005 Exposure to chronic stress downregulates corticosterone responses to acute stressors. Am. J. Physiol. Integr. Comp. Physiol. 288, R1628–R1636. (doi:10.1152/ajpregu.00484.2004) 10.1152/ajpregu.00484.2004 15886358

[34] Buchanan KL, Spencer KA, Goldsmith AR, Catchpole CK 2003 Song as an honest signal of past developmental stress in the European starling (*Sturnus vulgaris*). Proc. R. Soc. B 270, 1149–1156. (doi:10.1098/rspb.2003.2330) 10.1098/rspb.2003.2330 PMC1691349 12816653

[35] Wingfield JC 1994 Modulation of the adrenocortical response to stress in birds. In Perspectives in comparative endocrinology (eds Davey KG, Peter RE, Tobe SS), pp. 520–528. Ottawa: National Research Council Canada.

[36] R Core Team. 2016 R: A language and environment for statistical computing. Vienna, Austria: R Foundation for Statistical Computing.

[37] Bates D, Machler M, Bolker B, Walker S 2015 Fitting linear mixed-effects models using lme4. J. Stat. Softw. 67, 1–48.

[38] Andrews C et al. 2017 Data and script for ‘A marker of biological age explains individual variation in the strength of the adult stress response’ Zenodo Repository (https://doi.org/10.5281/zenodo.846830) 10.1098/rsos.171208 PMC5627134 28989794

[39] Viechtbauer W 2010 Conducting meta-analyses in *R* with the metafor package. J. Stat. Softw. 36, 1–48. (doi:10.18637/jss.v036.i03)

[40] Mazerolle MJ 2016 AICcmodavg: Model selection and multimodel inference based on (Q)AIC(c). R package version 2.0-4. (http://CRAN.R-project.org/package=AICcmodavg)

[41] Symonds MRE, Moussalli A 2010 A brief guide to model selection, multimodel inference and model averaging in behavioural ecology using Akaike's information criterion. Behav. Ecol. Sociobiol. 65, 13–21. (doi:10.1007/s00265-010-1037-6)

[42] Zimmer C, Spencer KA 2014 Modifications of glucocorticoid receptors mRNA expression in the hypothalamic-pituitary-adrenal axis in response to early-life stress in female Japanese quail. J. Neuroendocrinol. 26, 853–860. (doi:10.1111/jne.12228) 25303060 10.1111/jne.12228 PMC4260142

[43] Heidinger BJ, Nisbet ICT, Ketterson ED 2008 Changes in adrenal capacity contribute to a decline in the stress response with age in a long-lived seabird. Gen. Comp. Endocrinol. 156, 564–568. (doi:10.1016/j.ygcen.2008.02.014) 18378235 10.1016/j.ygcen.2008.02.014

[44] Tacutu R, Craig T, Budovsky A, Wuttke D, Lehmann G, Taranukha D, Costa J, Fraifeld VE, De Magalhães JP 2013 Human ageing genomic resources: integrated databases and tools for the biology and genetics of ageing. Nucleic Acids Res. 41, 1027–1033. (doi:10.1093/nar/gks1155) 10.1093/nar/gks1155 PMC3531213 23193293

[45] Williams G 1957 Pleiotropy, natural selection, and the evolution of senescence. Evolution 11, 398–411. (doi:10.1111/j.1558-5646.1957.tb02911.x)

[46] Kirkwood TBL 2005 Understanding the odd science of aging. Cell 120, 437–447. (doi:10.1016/j.cell.2005.01.027) 15734677 10.1016/j.cell.2005.01.027

[47] Love OP, McGowan PO, Sheriff MJ 2013 Maternal adversity and ecological stressors in natural populations: the role of stress axis programming in individuals, with implications for populations and communities. Funct. Ecol. 27, 81–92. (doi:10.1111/j.1365-2435.2012.02040.x)

[48] Crino OL, Driscoll SC, Breuner CW 2014 Corticosterone exposure during development has sustained but not lifelong effects on body size and total and free corticosterone responses in the zebra finch. Gen. Comp. Endocrinol. 196, 123–129. (doi:10.1016/j.ygcen.2013.10.006) 24188885 10.1016/j.ygcen.2013.10.006

[49] Kriengwatana B, Wada H, Schmidt KL, Taves MD, Soma KK, MacDougall-Shackleton SA 2014 Effects of nutritional stress during different developmental periods on song and the hypothalamic-pituitary-adrenal axis in zebra finches. Horm. Behav. 65, 285–293. (doi:10.1016/j.yhbeh.2013.12.013) 24417905 10.1016/j.yhbeh.2013.12.013

[50] Lendvai ÁZ, Loiseau C, Sorci G, Chastel O 2009 Early developmental conditions affect stress response in juvenile but not in adult house sparrows (*Passer domesticus*). Gen. Comp. Endocrinol. 160, 30–35. (doi:10.1016/j.ygcen.2008.10.004) 18955057 10.1016/j.ygcen.2008.10.004

[51] Love OP, Williams TD 2008 Plasticity in the adrenocortical response of a free-living vertebrate: the role of pre- and post-natal developmental stress. Horm. Behav. 54, 496–505. (doi:10.1016/j.yhbeh.2008.01.006) 18313054 10.1016/j.yhbeh.2008.01.006

[52] Bateson P, Gluckman P, Hanson M 2014 The biology of developmental plasticity and the predictive adaptive response hypothesis. J. Physiol. 592, 2357–2368. (doi:10.1113/jphysiol.2014.271460) 24882817 10.1113/jphysiol.2014.271460 PMC4048093

[53] Nettle D, Bateson M 2015 Adaptive developmental plasticity: what is it, how can we recognize it and when can it evolve? Proc. R. Soc. B 282, 20151005 (doi:10.1098/rspb.2015.1005) 10.1098/rspb.2015.1005 PMC4528519 26203000

[54] Monaghan P, Haussmann MF 2015 The positive and negative consequences of stressors during early life. Early Hum. Dev. 91, 643–647. (doi:10.1016/j.earlhumdev.2015.08.008) 26385447 10.1016/j.earlhumdev.2015.08.008 PMC4706554

[55] Nettle D, Frankenhuis WE, Rickard IJ 2013 The evolution of predictive adaptive responses in human life history. Proc. R. Soc. B 280, 20131343 (doi:10.1098/rspb.2013.1343) 10.1098/rspb.2013.1343 PMC3730599 23843395

[56] Herborn KA, Heidinger BJ, Boner W, Noguera JC, Adam A, Daunt F, Monaghan P 2014 Stress exposure in early post-natal life reduces telomere length: an experimental demonstration in a long-lived seabird. Proc. R. Soc. B 281, 20133151 (doi:10.1098/rspb.2013.3151) 10.1098/rspb.2013.3151 PMC3973262 24648221

[57] Hazard D, Couty M, Richard S, Guémené D 2008 Intensity and duration of corticosterone response to stressful situations in Japanese quail divergently selected for tonic immobility. Gen. Comp. Endocrinol. 155, 288–297. (doi:10.1016/j.ygcen.2007.05.009) 17586506 10.1016/j.ygcen.2007.05.009

[58] Redei EE 2008 Molecular genetics of the stress-responsive adrenocortical axis. Ann. Med. 40, 139–148. (doi:10.1080/07853890701724863) 18293144 10.1080/07853890701724863

